# Increased prevalence of thyroid dysfunction in Tehran – HAMRAH study

**DOI:** 10.1186/s12902-023-01524-x

**Published:** 2023-12-06

**Authors:** Zahra Ghaemmaghami, Parisa Firoozbakhsh, Delara Gholami, Sajad Khodabandelu, Mohammadreza Baay, Mohammad Javad Alemzadeh-Ansari, Bahram Mohebbi, Zahra Hosseini, Shabnam Boudagh, Hamidreza Pouraliakbar, Yeganeh Pasebani, Ali Rafati, Ehsan Khalilpour, Yasaman Khalili, Maedeh Arabian, Majid Maleki, Hooman Bakhshandeh, Parham Sadeghipour

**Affiliations:** 1 Rajaie Cardiovascular Medical and Research Center, Iran University of Medical Sciences, Vali-Asr Ave, Tehran, 1995614331 Iran; 2 Cardio-Oncology Research Center, Rajaie Cardiovascular Medical and Research Center, Iran University of Medical Sciences, Tehran, Iran; 3 Imam Khomeini Hospital Complex, Tehran University of Medical Sciences, Tehran, Iran; 4 Department of Biostatistics and Epidemiology, Student Research Committee, School of Health, Mazandaran University of Medical Sciences, Sari, Iran; 5 Cardiovascular Intervention Research Center, Rajaie Cardiovascular Medical and Research Center, University of Medical Sciences, Vali-Asr Ave, 1995614331 Tehran, Iran; 6 Echocardiography Research Center, Rajaie Cardiovascular Medical and Research Center, University of Medical Sciences, Tehran, Iran

**Keywords:** Hypothyroidism, Hyperthyroidism, Thyroid Diseases, Prevalence, Cohort studies, Iran

## Abstract

**Background:**

The aim of the current study is to assess the prevalence of different categories of thyroid dysfunction and their associated risk factors among the modern urban population of Tehran, the capital of Iran.

**Methods:**

The present investigation is a sub-study of the HAMRAH study, a population-based prospective study designed to assess the prevalence of traditional cardiovascular risk factors and their changes through a 10-year follow-up. 2228 (61% female) adults aged between 30 and 75 years old and with no overt cardiovascular diseases were selected through a multistage cluster randomized sampling. Blood levels of thyroid-stimulating hormone (TSH), thyroxin (T4), and triiodothyronine (T3) were measured with the aim of assessing the prevalence of abnormal thyroid function status among the modern urban Iranian population, and in order to report the total prevalence of participants with clinical hypo- or hyperthyroidism, the number of individuals taking thyroid-related drugs were added to the ones with overt thyroid dysfunction. A subgroup analysis was also performed to determine the associated risk factors of thyroid dysfunction.

**Results:**

The prevalence of thyroid dysfunction among the total population was 7% (95%CI: 5.9 − 8%) and 0.4% (95% CI: 0.1 − 0.6%) for subclinical and overt hypothyroidism, and 1.6% (95% CI: 1 − 2%) and 0.2% (95% CI: 0 − 0.3%) for subclinical and overt hyperthyroidism, respectively. Clinical thyroid dysfunction was detected in 10.3% of the study population (9.4% had clinical hypo- and 0.9% had clinical hyperthyroidism). In the subgroup analysis, thyroid dysfunction was significantly more prevalent among the female participants (*P*-value = 0.029).

**Conclusions:**

In the current study, the prevalence of different categories of abnormal thyroid status, and also the rate of clinical hypo- and hyperthyroidism was assessed using the data collected from the first phase of the HAMRAH Study. In this study, we detected a higher prevalence of clinical and subclinical hypothyroidism among the Iranian population compared to the previous studies.

**Supplementary Information:**

The online version contains supplementary material available at 10.1186/s12902-023-01524-x.

## Background

Thyroid dysfunction stands for the most common endocrine disorder after diabetes [1] and is representative of about 30–40% of the patients visited in an endocrine clinic [2]. The prevalence of thyroid dysfunction has been evaluated in multiple national and international studies. The National Health and Nutrition Examination Survey (NHANES III), the largest community-based study evaluating thyroid dysfunction in the United States, reported the prevalence of subclinical and overt hypothyroidism as 4.3% and 0.3%, respectively in 17,353 participants aged 12 years and older. Also, subclinical and overt hyperthyroidism was observed in 0.5% and 0.7% of the patients, respectively [3]. Based on a systematic review and meta-analysis performed on 17 relevant studies published from 1975 to 2012, the reported prevalence of hypo- and hyperthyroidism in Europe was 3.05% and 0.75%, respectively [4]. Similarly in large Iranian population-based cohorts, overt hypo- and hyperthyroidism was reported in 2-2.8% and 0.8-2.2% of the participants, and subclinical thyroid dysfunction was more prevalent than the overt form, with a frequency of 5.5-5.8% for subclinical hypo- and 0.9-3.7% for subclinical hyperthyroidism [5, 6].

Untreated thyroid abnormalities can cause serious complications including osteoporosis, neuropsychiatric problems, and dyslipidemia which can eventually lead to significant cardiovascular events such as coronary artery disease, heart failure, and even cardiovascular mortality risk [7, 8]. The rate of thyroid disorders varies widely in different subsets and is strongly associated with age, ethnicity, genetic predisposing factors, geographic and cultural determinants, as well as the iodine intake status of the studied population [9]. These socio-demographic varieties could change the face of the disease and have an important impact on public healthcare policies. For instance, after the introduction of the Universal Salt Iodization (USI) strategy, autoimmune thyroid disorders were detected more frequently in iodine-sufficient areas [10].

HAMRAH study is a population-based prospective study conducted with the aim of assessing the prevalence of cardiovascular risk factors in adult residents of Tehran, the capital of Iran, and evaluating their changes through a 10-year follow-up [11]. In the current sub-study of HAMRAH, the prevalence of thyroid abnormalities and their associated risk factors was reported in the modern urban Iranian population.

## Methods

### Study design & population

Heart Assessment and Monitoring in Rajaie Hospital (HAMRAH) is a population-based prospective study conducted at Rajaie Cardiovascular Medical and Research Center, the largest tertiary cardiovascular center in Iran. Participants were selected among fully informed residents of Tehran aged 30–75 years without any previous history of cardiovascular disease or cardiac intervention and with no communication skills defect, through a multistage cluster randomized sampling process. Two districts were randomly selected from each of the northern, southern, eastern, western, and central regions of Tehran, the capital of Iran. Then 60 clusters each including 20 households were selected through the proportional-to-size approach.

HAMRAH study consists of two consecutive phases: First, a cross-sectional phase was carried out as a population-based survey which was conducted from August 2017 to August 2019. In this phase, participants were assessed for the common cardiovascular disease (CVD) risk factors, laboratory indices, electrocardiography and echocardiography features, diet, physical activity levels, psychological aspects, and peripheral vascular diseases. The current sub-study is based on the data collected from this cross-sectional phase. Second, an ongoing longitudinal phase is carried out as a population-based prospective study and the participants will be followed for ten years, with 2-year interval follow-up visits. In this phase, the changes in the aforementioned factors and also the incidence of CVDs will be evaluated. A detailed description of the methodology and goals of the HAMRAH is previously published [11]. The study was approved by the ethics committee of the Rajaie cardiovascular medical and research center (RHC.AC.IR.REC.1396.22).

### Thyroid hormones measurements

The HAMRAH study’s participants were referred for laboratory tests after 8 h of fasting [11]. Serum TSH, total T4, and T3 levels were measured using the radioimmunoassay technique (Abbott Laboratories, North Chicago, Ill). According to the manufacturer’s instruction for use, the functional sensitivity for the TSH assay was ≤ 0.01mIU/L, and the limit of quantitation for the total T4 assay was ≤ 3.0 µg/dL. All assays were adjusted to the respective manufacturer’s performance designations. The laboratory reference range for TSH was 0.32–5.06 mIU/L (based on the reference range in the Iranian population) [12], and normal T4 levels were 4.87–11.72 µg/dL. Definitions of different types of thyroid dysfunction are presented in Supplementary Table S1.

### Statistical analysis

Data variables were described as frequency (percentage) for the categorical and mean (standard deviation) for the interval variables. The prevalence of different thyroid dysfunction categories was presented with their 95% CI. Blood levels of the thyroid hormones were determined via median and percentiles. Sub-group analysis was performed by Pearson’s chi-square and chi-square for trend tests, and *P* value < 0.05 was considered statistically significant. Multinomial logistic regression model was applied to assess the adjusted associations between the thyroid function states and the participants’ sex, age, BMI group, and cigarette smoking status. Due to the multistage randomized sampling method, correction for the design effect was applied to the descriptive and analytic statistical methods. Tehran districts were considered as the primary sampling units. Sampling weights were computed according to population and sample numbers of each district. Statistical analysis was conducted using Stata (StataCorp. 2015. Stata Statistical Software: Release 14. College Station, TX: StataCorp LP.)

## Results

A total of 2228 individuals (mean age: 49 ± 11.3 years, 1361 (61%) female) participated in the HAMRAH study, and their thyroid function test results were available for analysis in the current sub-study. The baseline characteristics of the participants are presented in Table 1.

**Table 1:** Baseline demographics and clinical characteristics of the participants

Variable	Descriptive Statistics
Age (years), mean (SD) [min – max]	49 (11.3) [30–75]
Age ≥ 50 years, n (%)	1044 (47%)
Sex, n (%)	
Female	1361 (61%)
Male	867 (39%)
BMI, mean (SD) [min – max]	29.1 (4.7) [11.1–47.9]
BMI category, n (%)	
Normal & under-weight (< 25)	378 (17%)
Overweight (25–29.9)	1038 (47%)
Obese (≥ 30)	812 (36%)
Diabetes Mellitus, n (%)	300 (14.3%)
Hypertension, n (%)	510 (27.4%)
Dyslipidemia, n (%)	402 (22.6%)
Current cigarette smoker, n (%)	219 (12%)

BMI: body mass index, SD: standard deviation

The blood levels of thyroid hormones based on the participants’ sex are described in Supplementary Table S2. In the total study population, the median (inter-quartile range) of serum TSH, T4, and T3 concentrations were 1.88 (1.25–2.8) mIU/L, 8.1 (7.2–9) µg/dL, and 1 (0.9–1.12) µg/dL, respectively. In Supplementary Figure S1, various percentiles for the blood levels of thyroid hormones are presented according to the different sex-age groups. Blood levels of thyroid hormones (including T3, T4, and TSH) were also measured in different BMI categories and reported as mean [95% CI] and median (IQR) in Supplementary Table S3.

### Thyroid functional status

The participants’ thyroid status, according to the diagnostic laboratory criteria mentioned in Table S1, is reported in Table 2. Abnormal thyroid status was detected in 9.2% of the total population (7% and 0.4% were in the subclinical and overt hypothyroid state, while 1.6% and 0.2% were in the subclinical and overt hyperthyroid state, respectively).

**Table 2:** – Prevalence of thyroid abnormalities in HAMRAH study

Thyroid Function Status	Frequency	Prevalence (95% CI)
Subclinical hypothyroidism	156	7% (5.9-8%)
Overt hypothyroidism	8	0.4% (0.1-0.6%)
Subclinical hyperthyroidism	35	1.6% (1-2%)
Overt hyperthyroidism	4	0.2% (0-0.3%)

95% CI: 95% confidence interval

10% of our study population had a history of using thyroid-related drugs, 204 individuals (9.2%) used levothyroxine, and 17 participants (0.8%) used methimazole. In order to report the total prevalence of participants with clinical hypo- or hyperthyroidism, participants taking thyroid-related drugs were added to the ones with overt thyroid dysfunction. By this method, 10.3% of the participants were detected with clinical thyroid dysfunction (9.4% had clinical hypothyroidism and 0.9% had clinical hyperthyroidism, respectively). The prevalence [95% CI] of different thyroid status categories in different sex-age groups and different BMI categories is demonstrated in Supplementary Table S4 and Table S5, respectively.

Among the 204 and 17 participants taking levothyroxine and methimazole, 166 (81.3%) and 14 (82.3%) were in the euthyroid state, respectively. More details about the thyroid status of the participants taking thyroid-related drugs are presented in Supplementary Table S6.

### Sub-group analysis

After combining the overt and sub-clinical states, the prevalence of hypo-, hyper-, and euthyroid conditions were described in different subgroups including sex, age (lesser or greater than 50 years old), BMI categories (normal, overweight, and obese), and smoking status and the results are presented in Table 3. It can be observed that different thyroid dysfunction states are more prevalent among females rather than males (*p*-value = 0.029).

**Table 3:** Thyroid dysfunction in different subgroups

	Euthyroid (n = 2025)	Hypothyroidism (n = 164)	Hyperthyroidism (n = 39)	***P*** value
**Sex**				0.029
Female	1220 (60.2%)	112 (68.3%)	29 (74.4%)	
Male	805 (39.8%)	52 (31.7%)	10 (25.6%)	
**Age**				0.22
<50	1088 (53.7%)	78 (47.6%)	18 (46.2%)	
≥50	937 (46.3%)	86 (52.4%)	21 (53.8%)	
**BMI**				0.301
underweight and normal	347 (17.1%)	24 (14.6%)	7 (17.9%)	
overweight	952 (47%)	66 (40.2%)	20 (51.3%)	
obese	726 (35.9%)	74 (45.1%)	12 (30.8%)	
**Cigarette Smoking**				0.132
Yes	255 (12.6%)	12 (7.3%)	6 (13.4%)	
No	1770 (87.4%)	152 (92.7%)	33 (84.6%)	

BMI: body mass index. *P*-value less than 0.05 is considered significant

Multinomial logistic regression was also performed according to the mentioned factors and the results are demonstrated in Supplementary Table S7. According to the multinomial logistic regression, no statistically significant association was detected between these factors and the thyroid status.

## Discussion

In this sub-study of the HAMRAH study, we assessed the prevalence of thyroid dysfunction among 2228 adults who were randomly selected from the modern urban population of Iran. Comparing to previous similar large scale cohorts, we detected a higher prevalence of clinical and subclinical hypothyroidism, in addition to lower prevalence of overt hyperthyroidism.

The prevalence of thyroid disorders in Iran was previously assessed in two large cohort studies. Tehran Thyroid Study (TTS) was conducted on 5769 subjects who were selected from only one district of Tehran between March 1997 and December 2004 [13]. Isfahan Thyroid Cohort Study (ITCS) is another population-based cohort study that was designed in 3 phases and was performed on 2523 subjects selected from urban areas of Isfahan. The first phase was conducted from 2006 to 2011 to evaluate the prevalence of thyroid disorders [14]. Thus, despite their large sample size, recruitment was performed between 10 and 25 years ago. Since the country has faced various socio-economic challenges, mentioned studies might not present the actual state of thyroid diseases. In HAMRAH study recruitment was performed between August 2017 and August 2019, and participants were selected among more modern urban population using random sampling over the entire city and consequently, the study can possibly represent a more unbiased picture of the diseases. Although the prevalence of thyroid dysfunction varies in different regions according to the age, ethnicity, genetic background, geographical, environmental and cultural factors, and iodine intake status of the individuals, the additional factors that can justify the observed difference in the prevalence of hypothyroidism in our study compared to previous ones include our multistage cluster randomized sampling process and the more recent conductance of our study that makes our findings a better and more updated representative of Iranian population’s thyroid status.

The prevalence of different forms of thyroid dysfunction in our study and previous large Iranian population-based cohorts is demonstrated and compared in Table 4. Overally, we have observed a higher rate of subclinical hypothyroidism compared to the national and international cohorts.

**Table 4:** Comparing the results of HAMRAH study with previous studies

	Thyroid Functional Status			
Studies	Subclinical Hypothyroidism	Overt Hypothyroidism	Subclinical Hyperthyroidism	Overt Hyperthyroidism
HAMRAH study	7%	0.40%	1.60%	0.20%
TTS study	5.50%	2%	3.70%	2.20%
ITCS study	5.80%	2.80%	0.99%	0.80%
Meta-analysis of hyperthyroidism in Iran	-	-	1.52%	0.69%

HAMRAH: Heart Assessment and Monitoring in Rajaie Hospital, TTS: Tehran Thyroid Study, ITCS: Isfahan Thyroid Cohort Study

Subclinical thyroid dysfunction can negatively affect cardiovascular health, skeletal system, cognitive function, and is also associated with reduced quality of life. Previous studies have also demonstrated that hypothyroidism, both in its overt and subclinical forms, can increase the risk of metabolic syndrome and its components, including abdominal obesity and hypertriglyceridemia, especially among elderlies [15]. A positive association was also detected between serum levels of free T4 and blood pressure profiles, including systolic and diastolic blood pressure, pulse pressure, and mean arterial pressure [16]. Combination of thyroid dysfunction and metabolic syndrome or its components, can negatively influence the individual’s cardiovascular health, especially among the aged population. Despite the potential risk of progression of subclinical hypothyroidism to the overt form which can have a varying rate of 1.76–73.47 cases per 100 patient-years base on the baseline TSH, there are controversies regarding its management and defining the optimal cut-off value of thyroid hormones in which the treatment should be started [17, 18]. Considering the mentioned rate of transformation to the clinical form of the disease, this increase in subclinical hypothyroidism warrants careful healthcare policy planning and structural follow up program. The pooled analysis of Sajjadi-Jazi et al. in a systematic review of 12 studies and 37,883 Iranian participants showed that subclinical hyperthyroidism was not different in HAMRAH compared to previous studies, proving the fact that subclinical hyperthyroidism followed a more stable course during the recent years [9]. Overt forms of both hypo- and hyperthyroidism were observed less frequently in HAMRAH population, potentially because 10% of the participants had a history of receiving thyroid-related drugs. After adding the participants with a history of receiving levothyroxine to the ones with overt hypothyroidism, HAMRAH study detected a higher rate of clinical hypothyroidism (9.4%) compared to previous Iranian studies. Although during the study period, Tehran and Isfahan were both iodine sufficient for many years through salt fortification with iodine, these epidemiological differences can be justified by variations in definitions of thyroid abnormalities, inclusion criteria and study population of the studies, and the influence of age, gender and environmental factors [19].

10% of our study population was receiving thyroid-related drugs. Among the participants taking levothyroxine, 81.3% were in the euthyroid state, 10.8% were still hypothyroid and 7.9% of them were in the hyperthyroid state, and among patients receiving methimazole, 82.3% became euthyroid, 5.9% had subclinical hypothyroidism and 11.8% of them were still in the hyperthyroid state, while American studies reported lower rates of receiving proper treatment. The prevalence of euthyroid patients among those receiving thyroid medications was 60.1% in the Colorado study and 66.7% in the NHANES III study [3, 20]. Although we have observed a more appropriate thyroid management in our cohort, still around 20% of patients are mistreated. Untreated/mistreated thyroid dysfunction can have serious physical complications, including cardiovascular disease, osteoporosis, and infertility [21]. Thyroid dysfunction is also associated with a significant economic burden (estimated between $460 and $2555 per patient per year) [22], and an increase in the rate of sick leave and absenteeism from work and can negatively influence the employment rate and working ability of the subjects [23], all highlighting the importance of adjusting the thyroid drug dosage for each individual and keeping them in the euthyroid state, and also the essential role of having a regular screening plan for early detection of thyroid dysfunction.

In HAMRAH Study, all types of thyroid dysfunction were detected more frequently among the female population except for overt hypothyroidism which was more prevalent in the male population (1.86% vs. 1.21%), while in ITCS, all thyroid dysfunction phenotypes were more common in women than men [14]. We also performed a subgroup analysis to report the prevalence of thyroid dysfunction according to the participants’ sex, age, BMI, and smoking status. According to the subgroup analysis, the rate of thyroid dysfunction was significantly higher among the female population, but this association didn’t remain statistically significant in the multinomial logistic regression. There was no statistically significant association between other factors and thyroid status in both subgroup analysis and multinomial logistic regression. In the previous studies, the female sex was also recognized as an established risk factor for thyroid dysfunction, which confirms our findings [24].

In the current study, no significant association was detected between participants’ smoking status and their thyroid function state. Multiple studies have detected a negative association between smoking and hypothyroidism and a positive association between smoking and hyperthyroidism. Smoking can also reversibly decrease the serum levels of thyrotropin hormone [25, 26]. In our study, although serum TSH and T4 were higher in overweight and obese individuals compared with normal and underweight ones, the difference between the groups was not statistically significant. A cross-sectional survey was performed on 5353 individuals in the framework of the Tehran thyroid Study (TTS) with the aim of investigating the association of BMI and thyroid function status. This study revealed that obesity is associated with a higher prevalence of hypothyroidism, both overt and subclinical, and higher rates of TPOAb positivity [27]. NHANES III Study also reported a positive association between participants’ BMI and waist circumference and the serum TSH and FT3, but not FT4.

The current sub-study had several limitations. First, the primary goal of this prospective study was to evaluate the prevalence of cardiovascular risk factors among healthy adult residents of Tehran and since the main focus of the current study was cardiovascular diseases, data regarding several specific risk factors of thyroid disorders (including the history of other autoimmune diseases like pernicious anemia or celiac disease, family history of thyroid disorder, and previous history of radiation to the neck or upper chest) were not collected. Second, in order to report the prevalence of clinical thyroid dysfunction, we considered ones receiving levothyroxine or methimazole as patients with overt thyroid dysfunction and added them to those with overt hypo- or hyperthyroid state in their lab results. Since levothyroxine can be prescribed for other purposes than overt hypothyroidism, like subclinical thyroid dysfunction, non-toxic multinodular goiter, or thyroid nodule, we may have overestimated the prevalence of clinical hypothyroidism in our evaluation [28]. Third, although the random sampling method was used for recruitment in the current study, the sample population might not fully represent the general population. Finally, due to resource limitations, the anti-TPO antibody was not measured for determining the autoimmune background of the thyroid dysfunction and an ultrasound examination of the thyroid gland was not performed for assessing thyroid nodules or goiter in this study.

## Conclusions

In conclusion, we assessed the prevalence of different categories of abnormal thyroid status, and also the rate of clinical hypo- and hyperthyroidism using the data collected from the first phase of the HAMRAH Study. In the current study, a higher prevalence of clinical and subclinical hypothyroidism was detected among the Iranian population compared to the previous studies.

### Electronic supplementary material

Below is the link to the electronic supplementary material.

Supplementary Material 1

## Data Availability

The datasets used and analyzed during the current study are available from the corresponding authors on reasonable request.

## Abbreviations

USI: Universal Salt Iodization
NHANES III: National Health and Nutrition Examination Survey
HAMRAH: Heart Assessment and Monitoring in Rajaie Hospital
CVD: cardiovascular disease
TSH: thyroid stimulating hormone
T4: thyroxine
T3: Triiodothyronine
SD: standard deviation
CI 95%: confidence interval 95%
IQR: inter-quartile range
BMI: Body mass index
TTS: Tehran Thyroid Study
ITCS: Isfahan Thyroid Cohort Study
anti-TPO antibody: anti-thyroid peroxidase antibody
