# Supplementary material for: Increased prevalence of thyroid dysfunction in Tehran – HAMRAH study
Source: BMC Endocr Disord. 2023 Dec 6;23:270. doi: 10.1186/s12902-023-01524-x (PMC10698901; doi:10.1186/s12902-023-01524-x)
Supplement: Supplementary file 1 — Supplementary Material 1 [file 12902_2023_1524_MOESM1_ESM.docx]

**Supplementary files**

**Increased Prevalence of Thyroid Dysfunction in Tehran– HAMRAH study**

**Table of contents:**

- **Methods:**

Table S1…………………………………………………………………………………… Page 2

- **Results:**

Table S2 ………………………………………………………………………………….... Page 3

Table S3 …………………………………………………………………………………… Page 4

Table S4 …………………………………………………………………………………… Page 5

Table S5 …………………………………………………………………………………… Page 6

Table S6 …………………………………………………………………………………… Page 7

Table S7 …………………………………………………………………………………… Page 8

Figure S1 …………………………………………………………………………………... Page 9

| **Table S1:** Definition of different types of thyroid dysfunction applied in HAMRAH study | |
| --- | --- |
| **Type of thyroid dysfunction** | **Definition** |
| Subclinical hypothyroidism | TSH>5.06 mIU/L and normal T4 values |
| Overt hypothyroidism | TSH>5.06 mIU/L and T4<4.87 μg/dL |
| Subclinical hyperthyroidism | TSH<0.32 mIU/L and normal T4 values |
| Overt hyperthyroidism | TSH<0.32 mIU/L and T4>11.72 μg/dL |
| TSH: thyroid stimulating hormone, T4: thyroxine | |

| **Table S2 –** Blood levels of thyroid hormones based on the sex of the participants | | | | | | | | |
| --- | --- | --- | --- | --- | --- | --- | --- | --- |
|  | **T3** | |  | **T4** | |  | **TSH** | |
| **Sex** | **Mean  [CI95%]** | **Median  (Q1 – Q3)** |  | **Mean  [CI95%]** | **Median  (Q1 – Q3)** |  | **Mean  [CI95%]** | **Median  (Q1 – Q3)** |
| **Female** | 1.06  [0.96 - 1.15] | 1  (0.89 - 1.11) |  | 8.49  [8.04 - 8.93] | 8.18  (7.4 - 9.21) |  | 2.81  [2.42 - 3.21] | 1.9  (1.31 - 3.1) |
| **Male** | 1.07  [0.97 - 1.16] | 1  (0.91 - 1.13) |  | 7.84  [7.59 - 8.1] | 7.9  (6.97 - 8.63) |  | 2.29  [2.06 - 2.53] | 1.77  (1.17 - 2.3) |
| **Total** | 1.06  [0.97 - 1.16] | 1  (0.9 - 1.12) |  | 8.26  [7.91 - 8.61] | 8.1  (7.2 - 9) |  | 2.63  [2.29 - 2.97] | 1.88  (1.25 - 2.8) |

T3: Triiodothyronine, T4: thyroxine, TSH: thyroid stimulating hormone, CI95%: confidence interval 95%

| **Table S3 -** Blood levels of thyroid hormones in different BMI categories | | | | | | | | |
| --- | --- | --- | --- | --- | --- | --- | --- | --- |
|  | **T3** | |  | **T4** | |  | **TSH** | |
|  | **Mean  [CI 95%]** | **Median  (Q1-Q3)** |  | **Mean  [CI 95%]** | **Median  (Q1-Q3)** |  | **Mean  [CI 95%]** | **Median  (Q1-Q3)** |
| **Underweight  and Normal**  **(BMI** < **25)** | 1.06  [0.91-1.2] | 1  (0.88-1.12) |  | 8.21  [7.89-8.53] | 8.04  (7.2-9) |  | 2.39  [1.85-2.92] | 1.88  (1.14-2.79) |
| **Overweight**  **(BMI = 25-29.9)** | 1.07  [0.97-1.16] | 1  (0.9-1.13) |  | 8.23  [7.87-8.6] | 8.1  (7.19-8.94) |  | 2.65  [2.11-3.18] | 1.88  (1.25-2.7) |
| **Obese**  **(BMI ≥ 30)** | 1.05  [0.98-1.12] | 1  (0.9-1.12) |  | 8.3  [7.89-8.71] | 8.1  (7.2-9.125) |  | 2.71  [2.26-3.16] | 1.88  (1.33-2.92) |

BMI: body mass index, T3: Triiodothyronine, T4: thyroxine, TSH: thyroid stimulating hormone, CI95%: confidence interval 95%

| **Table S4 –** Prevalence [CI95%] of observed thyroid status in various sex-age groups | | | | | | | |
| --- | --- | --- | --- | --- | --- | --- | --- |
| **Sex** | **Age group** |  | **Euthyroid** | **Overt  Hypothyroidism** | **Sub-clinical  Hypothyroidism** | **Overt  Hyperthyroidism** | **Sub-clinical  Hyperthyroidism** |
| **Male** | 30-39 (n=210) | n (%) | 199 (94.76%) | 0 (0%) | 8 (3.81%) | 0 (0%) | 3 (1.43%) |
|  |  | [CI95%] | [87,96.49]% |  | [1.214,10.61]% |  | [.9561,10.11]% |
|  | 40-49 (n = 243) | n (%) | 221 (90.95%) | 1 (0.41%) | 19 (7.82%) | 0 (0%) | 2 (0.82%) |
|  |  | [CI95%] | [82.52,95.9]% | [.0173,2.204]% | [3.579,15.75]% |  | [.1431,4.401]% |
|  | 50-59 (n = 205) | n (%) | 192 (93.66%) | 1 (0.49%) | 9 (4.39%) | 1 (0.49%) | 2 (0.98%) |
|  |  | [CI95%] | [75.62,96.22]% | [.0972,7.051]% | [2.358,20.18]% | [.0259,3.632]% | [.1922,13.49]% |
|  | >=60 (n = 209) | n (%) | 193 (92.34%) | 2 (0.96%) | 12 (5.74%) | 0 (0%) | 2 (0.96%) |
|  |  | [CI95%] | [85.05,96.3]% | [.1805,3.345]% | [3.332,10.64]% |  | [.1805,3.345]% |
| **Female** | 30-39 (n = 354) | n (%) | 327 (92.37%) | 1 (0.28%) | 20 (5.65%) | 1 (0.28%) | 5 (1.41%) |
|  |  | [CI95%] | [88.78,95.6]% | [.0111,1.487]% | [3.358,9.667]% | [.0111,1.487]% | [.4337,2.671]% |
|  | 40-49 (n = 377) | n (%) | 341 (90.45%) | 1 (0.27%) | 28 (7.43%) | 0 (0%) | 7 (1.86%) |
|  |  | [CI95%] | [84.27,92.34]% | [.0094,1.45]% | [6.272,14.15]% |  | [.4993,4.118]% |
|  | 50-59 (n = 374) | n (%) | 328 (87.7%) | 1 (0.27%) | 35 (9.36%) | 2 (0.53%) | 8 (2.14%) |
|  |  | [CI95%] | [78.66,92.52]% | [.0427,2.3]% | [4.342,16.98]% | [.2821,4.984]% | [1.169,5.636]% |
|  | >=60 (n = 256) | n (%) | 224 (87.5%) | 1 (0.39%) | 25 (9.77%) | 0 (0%) | 6 (2.34%) |
|  |  | [CI95%] | [81.32,87.58]% | [.1276,10.6]% | [9.909,13.74]% |  | [.8826,6.283]% |

CI95%: confidence interval 95%

| **Table S5 -** Prevalence of thyroid dysfunction in different BMI categories | | | | | |
| --- | --- | --- | --- | --- | --- |
| **BMI category** | **Euthyroid** | **Overt  Hypothyroidism** | **Sub-clinical  Hypothyroidism** | **Overt  Hyperthyroidism** | **Sub-clinical  Hyperthyroidism** |
| **Underweight  and Normal n(%)** | 328 (17.625) | 1 (4.782) | 23 (13.69) | 6 (15.56) | 359 (17.23) |
| **Overweight n(%)** | 820 (44.45) | 4 (65.3) | 60 (37.56) | 18 (60.27) | 904 (44.33) |
| **Obese  n(%)** | 665 (37.93) | 3 (29.92) | 70 (48.75) | 11 (24.17) | 750 (38.44) |
| **Total (n)** | 1813 | 8 | 153 | 35 | 2013 |

BMI: body mass index. The definition of different BMI categories is as follows: Underweight and normal: BMI <25, Overweight: BMI= 25-29.9, Obese: BMI ≥30

| **Table S6 –** Observed thyroid functional status in participants using thyroid-related drugs | | | | | |
| --- | --- | --- | --- | --- | --- |
| **Thyroid drug** | **Euthyroid** | **Overt Hypothyroidism** | **Sub-clinical Hypothyroidism** | **Overt Hyperthyroidism** | **Subclinical Hyperthyroidism** |
| **Levothyroxine (n=204)** | 166 (81.3%) | 2 (1%) | 20 (9.8%) | 1 (0.5%) | 15 (7.4%) |
| **Methimazole (n=17)** | 14 (82.3%) | 0 (0%) | 1 (5.9%) | 1 (5.9%) | 1 (5.9%) |

| **Table S7-** Multinomial logistic regression | | | | |
| --- | --- | --- | --- | --- |
|  | **Hypothyroidism  (Compared to euthyroidism)** | | **Hyperthyroidism  (Compared to euthyroidism)** | |
| **Variables** | **OR (CI 95%)** | **P-value** | **OR (CI 95%)** | **P-value** |
| Sex | 0.88 (0.6 - 1.3) | 0.54 | 0.47 (0.2 - 1.1) | 0.08 |
| Age (Greater or lesser than 50) | 1.2 (0.9 - 1.7) | 0.24 | 1.5 (0.8 - 3) | 0.21 |
| BMI group | 1.2 (1 - 1.6) | 0.1 | 0.92 (0.6 - 1.5) | 0.76 |
| Cigarette smoking | 0.56 (0.3 - 1.1) | 0.11 | 1.6 (0.6 - 4.6) | 0.36 |
| OR: odds ratio, BMI: body mass index. P-value less than 0.05 is considered significant | | | | |


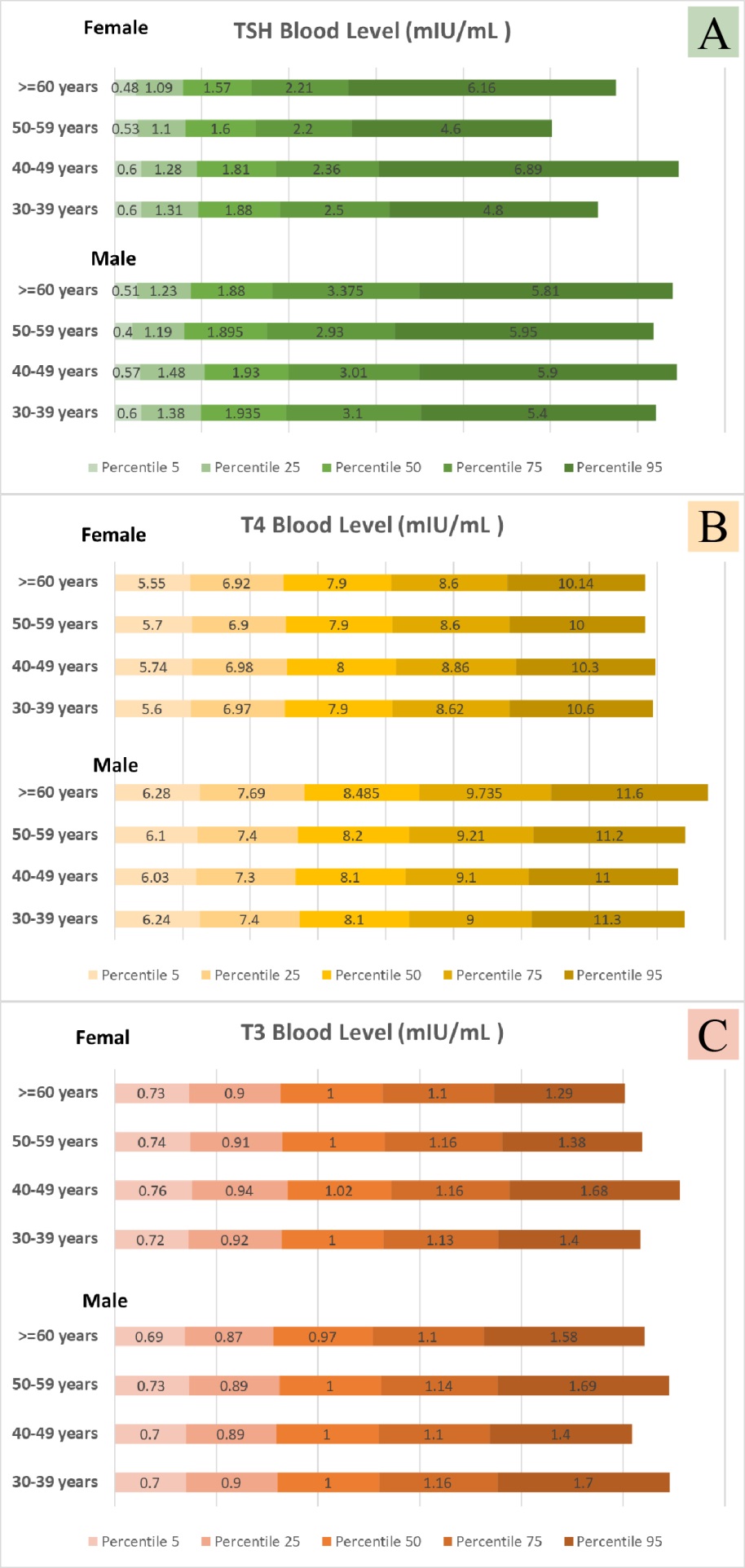


**Figure S1**- Percentiles for the blood levels of thyroid hormones, TSH (A), T4 (B), T3 (C) according to the different sex-age groups. TSH: thyroid stimulating hormone, T4: thyroxine, T3: Triiodothyronine
